# A personalized FEM model for reproducible measurement of anti-inflammatory drugs in transdermal administration to knee

**DOI:** 10.1038/s41598-021-04718-2

**Published:** 2022-01-13

**Authors:** Pasquale Arpaia, Federica Crauso, Mirco Frosolone, Massimo Mariconda, Simone Minucci, Nicola Moccaldi

**Affiliations:** 1 Laboratory of Augmented Reality for Health Monitoring (ARHeMLab), Department of Electrical Engineering and Information Technology, University of Naples Federico II, Naples, Italy; 2 Interdepartmental Center for Research in Health Management and Innovation in Health (CIRMIS), University of Naples Federico II, Naples, Italy; 3 Department of Advanced Biomedical Sciences, University of Naples Federico II, Naples, Italy; 4 Department of Public Health, University of Naples Federico II, Naples, Italy; 5 Department of Economics, Engineering, Society and Business Organization (DEIM), University of Tuscia, Viterbo, Italy

**Keywords:** Biomedical engineering, Drug delivery

## Abstract

A personalized model of the human knee for enhancing the inter-individual reproducibility of a measurement method for monitoring Non-Steroidal Anti-Inflammatory Drugs (NSAIDs) after transdermal delivery is proposed. The model is based on the solution of Maxwell Equations in the electric-quasi-stationary limit via Finite Element Analysis. The dimensions of the custom geometry are estimated on the basis of knee circumference at the patella, body mass index, and sex of each individual. An optimization algorithm allows to find out the electrical parameters of each subject by experimental impedance spectroscopy data. Muscular tissues were characterized anisotropically, by extracting Cole–Cole equation parameters from experimental data acquired with twofold excitation, both transversal and parallel to tissue fibers. A sensitivity and optimization analysis aiming at reducing computational burden in model customization achieved a worst-case reconstruction error lower than 5%. The personalized knee model and the optimization algorithm were validated in vivo by an experimental campaign on thirty volunteers, 67% healthy and 33% affected by knee osteoarthritis (Kellgren–Lawrence grade ranging in [1,4]), with an average error of 3%.

## Introduction

Transdermal administration of non-steroidal anti-inflammatory drugs, commonly known as NSAIDs, is widely used for treating knee joint inflammation. Usually, intramuscular infiltrations or less-invasive transdermal procedures (e.g. ionophoresis)^1^ are carried out.

To date, however, standardized methods for measuring the drug actually transmitted during the treatment are missing in transdermal delivery and, therefore, personalized medicine approach is prevented^2^. In the experimental campaign of Spear et al.^3^, only less than 60% of the therapies turned out to be effective. Conditions of different permeability of the tissues due to both inter-individual (e.g., age, sex, ethnicity) and intra-individual factors (e.g., different parts of the body, skin diseases, emotional states) prevent from verifying the therapy effectiveness^4^. This limits the clinical use of less-invasive transdermal delivery^5^. In fact, the drug actually administered by transdermal delivery in current clinical practice is assessed empirically, according to the operator experience^6^. In the case of local/regional drug therapy, the lack of scientific methods recognized and codified in shared procedures for in-vivo measurements involves significant issues for the definition of the *bioavailability*. The concept of bioavailability (rate of presence in blood or in urea after a dosage)^7,8^ is fundamental to establish the drug bioequivalence. However, it is not usable whenever the drug therapy is not systemic. For this reason, the equivalence between drugs is fundamentally based on the coincidence of the used active ingredient in the case of many current topical medications. However, the fundamental function of carriers and drug chemical formula in ensuring the absorption efficiency (and therefore bioavailability) is neglected in this way. In this framework, the European Medicine Agency recently underlined the urgency of defining and adopting new methods for the in-vivo assessment of local/regional drug therapies^4^.

From the electrical point of view, the introduction of a reference amount of conductive drug into a given volume of biological tissue produces a variation in the equivalent bioimpedance^9^. In previous studies^10,11^, the Drug Under Skin Meter (DUSM) was proposed to measure this impedance and so to estimate the amount of substance present in the subcutaneous tissues. In these experimental campaigns, issues related to interindividual reproducibility arose. Indeed, each tissue exhibits a specific electric behaviour because of its dimensions, shape, and electrical properties. It resulted in different responses of different tissues to the same drug amount administration in terms of impedance variation.

In this paper, the reproducibility uncertainty is faced by means of a numerical electrical model of the knee and an optimization algorithm to calculate the pre-infiltrated knee conditions for each individual. Then, these conditions are used as a sound basis for the identification of a personalized relationship between drug and impedance.

This paper is structured as follows: after a background on anatomical structure of the knee and its dielectric characterization in “Background” section, “Knee model personalization proposal” section reports the measurement production diagram and details the underlying numerical model of the knee. “Experimental validation” section reports the experimental campaign on human volunteers, the validation of the personalized model, and its customization on some selected volunteers.

## Background

### Knee structure

**Figure 1:**
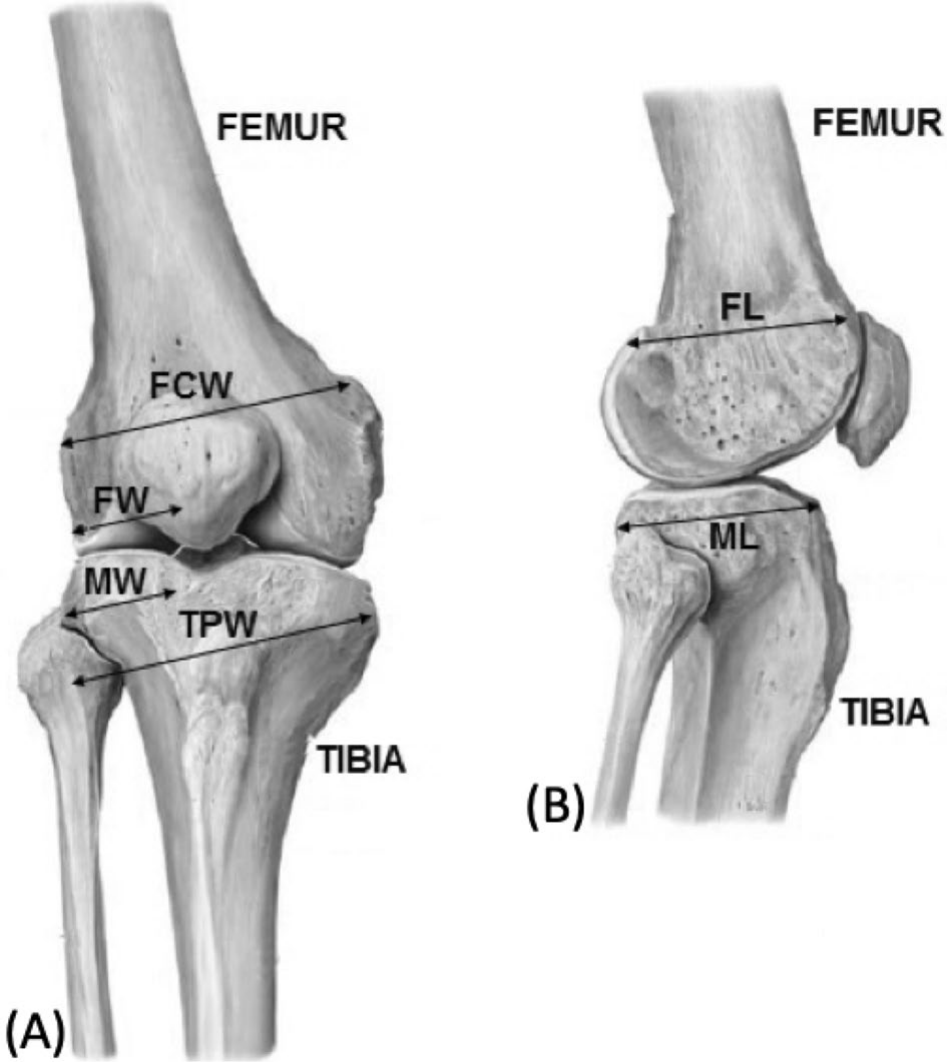
Knee bones dimensions, (**A**) anterior and (**B**) lateral view^13^: Femur Condyle width (FCW), medial Femur Width (FW), tibial plateau Medial Width (MW), Tibial Plateau Width (TPW), medial Femoral condyle Length (FL), tibial plateau Medial Length (ML).

The main bone components of the knee are: patella, tibia, and femur. Patella is a protruding bone, located in the front part of the knee joint. Tibia is a bulky bone placed in the lower part of the leg. Femur rests on it, namely located in the thigh, which is also part of the hip and knee. In^12^, tibia and femur dimensions were measured on 118 subjects by magnetic resonance imaging. In particular, average values of Plateau Width (TPW), plateau Medial Width (MW), and plateau Medial Length (ML) were measured for the tibia (Fig. 1A), and the Condyle width (FCW), medial Width (FW), and medial condyle Length (FL) were assessed for the femur (Fig. 1B). In Table 1, knee bones dimensions (average and standard deviation), are shown. The average values in male subjects are about 13–15% higher than in female ones.

**Table 1:** Typical tibia and femur dimensions for male and female.

	Bones (mm)	General	Male	Female
Tibia	TPW	74.9 ± 6.5	80.6 ± 3.9	69.5 ± 3.0
	ML	45.1 ± 4.5	47.9 ± 4.2	42.2 ± 2.9
	MW	30.4 ± 3.0	32.8 ± 2.1	28.1 ± 1.4
Femur	FCW	78.7 ± 6.7	84.2 ± 4.3	73.4 ± 3.6
	FL	52.2 ± 4.9	54.3 ± 4.8	50.2 ± 4.1
	FW	28.9 ± 3.3	30.6 ± 3.4	27.3 ± 2.6

The main muscles of knee are: (i) the quadriceps (upper part), a large fleshy muscle group covering the front and sides of the thigh, (ii) the gastrocnemius, a superficial muscle covering the entire posterior compartment of the leg^14^, and (iii) the soleus, located deeply relative to the latter^15^. Their thickness strongly depends on the individual morphotype and on the individual muscular tone.

The skin is the outermost layer of the knee. It is a keratinized multi-layered epithelial tissue consisting of epidermis, dermis, and hypodermis^16^. The epidermis is the outermost skin layer, structured in substrates as well: stratum corneum, lucidum, granulosum, spinosum, and basale^17^. The skin thickness depends on several factors including the explored body areas^18,19^: epidermis thickness ranges between 20–300 µm^20^, whilst dermis thickness ranges between 1–7 mm and differs for males and females^16,18^.

The hypoderm is a subcutaneous adipose tissue. The Subcutaneous Fat Thickness (SFT) depends on the body district and the physical subject conditions.

### Dielectric modeling of biological tissues

Dielectric properties of biological tissues in the frequency domain are characterized by ohmic and dielectric losses and can be modeled by means of a complex and frequency-dependent dielectric function, the Effective Dielectric Permittivity. Among the many state-of-the-art models of dielectric relaxations, the so called Cole–Cole equation was adopted, since it is widely used to model the dielectric properties of biological tissues. It expresses the Effective Dielectric Permittivity as (in the following, the subscript “r” standing for “relative” permittivity drops for the sake of the simplicity):

1 $$\begin{aligned} \begin{aligned} {\dot{\varepsilon }}_{eff}(\omega )=\varepsilon _{\infty }+\sum \limits _{n}\frac{\Delta \varepsilon _{n}}{1+(j\omega \tau _{n})^{1-\alpha _{n}}}+\frac{\sigma _{dc}}{j\omega \varepsilon _{0}} \end{aligned} \end{aligned}$$

where

- $\varepsilon _{\infty }$ is the value of permittivity in a frequency range high enough to consider the dielectric as unrelaxed;
- $\tau _n$ is the characteristic relaxation time, necessary for the material molecules or dipoles to return to the relaxed state after the application of the electric field;
- $\alpha _n$ is a coefficient affecting the flatness of the frequency spectrum of the n-th relaxation phenomenon;
- $\Delta \varepsilon _n$ is the difference between $\varepsilon _{s}$ (the static permittivity) and $\varepsilon _{\infty }$ in relation to the $n-th$ relaxation phenomenon;
- $\sigma _{dc}$ is the static conductivity;
- $\varepsilon _{0}$ the vacuum permittivity;
- and $\omega$ the angular frequency.

Several studies were carried out for the electrical characterization of biological tissues^21,22^. In^23^, skin, fat, muscle, and bone were analyzed. In^20,24^, the forearm skin was characterized on human volunteers by impedance spectroscopy. However, no specific knee study has ever been conducted. The most extensive and in-depth electrical characterization works on biological tissues are certainly those developed by Gabriel^25–27^.

## Knee model personalization proposal

**Figure 2:**
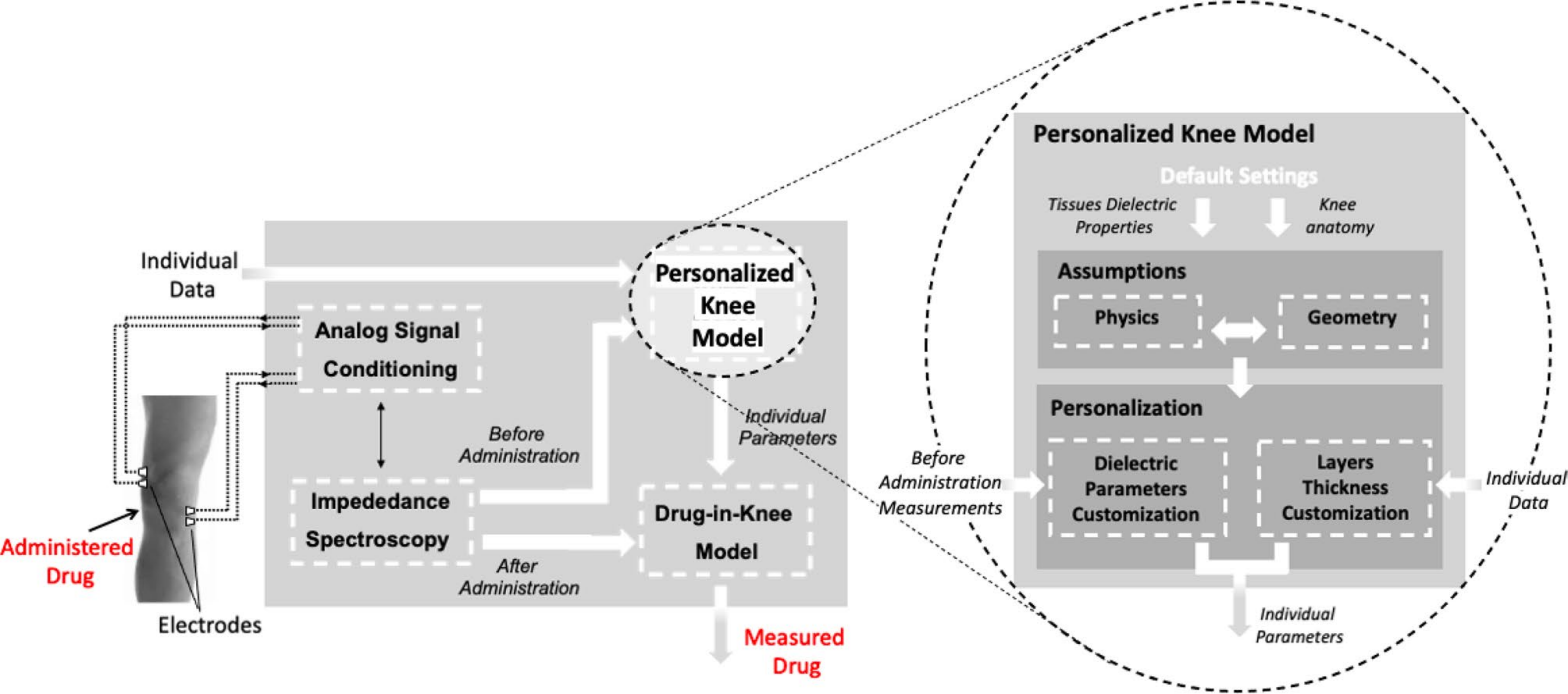
Diagram of measurement production process and particular of the Knee Model.

A numerical model of the knee joint based on “Finite Element Method” is proposed as the core of the measurement production process in NSAIDs transdermal delivery. The diagram of the measurement production is illustrated in Fig. 2. *Impedance Spectroscopy* is carried out by generating sinusoidal current spanning over a frequency range and by acquiring the voltage drop across the *Electrodes* and the *Analog Signal Conditioning* stage. A first measurement preceding the drug administration and *Individual Data* (i.e., sex, BMI, and knee circumference) are required in input by the *Personalized Knee Model* for the production of individual parameters. During drug administration, the relative change in impedance spectroscopy is measured and employed by the *Drug-in-Knee Model* (calibrated by the individual parameters of the *Personalized Knee Model*) for assessing the drug amount (*Measured Drug*).

The proposed model aims at identifying both healthy and osteoarthritic knees, but does not account for edema effusions. Indeed, literature confirms that a knee inflammation condition has a negligible impact on impedance measurements^28^. What above does not hold for edemas, due to their high ionic conductivity with respect to other surrounding tissues, especially whenever they are not minimal^29^.

In this section, (i) the *Assumptions* of the model and (ii) the *Personalization* procedure, are illustrated.

### Default settings

All tissues mentioned in “Background” section but the muscles can be described by means of a unique dielectric function. Muscle tissues of knee junction are skeletal muscles. This type of tissue consists of muscle fibers and fascicles (multiple bundles) of many cells joined together: muscle fibers of a fascicle are mutually parallel, but the fascicles may have variable direction, even different from tendons'^30^. These physiological characteristics immediately point out that skeletal muscles have anisotropic biologic properties, depending on the orientation of the muscle fibers^31–33^. Therefore, muscles are expected to show also different dielectric properties in two directions, along and perpendicular to their fibers. Indeed, literature surveys confirm such assumption, as reported by C. Gabriel where different frequency spectra for the Effective Dielectric Permittivity along the two directions are shown^34^. Literature data provide values and expression of the Effective Dielectric Permittivity functions for all the knee joint tissues but the muscles. For them, the only provided dielectric description is with an external electric excitation field perpendicular to muscular fibers.

A more detailed muscles model has been achieved by an identification procedure set up starting from the experimental data of the Effective Dielectric Permittivity function available in^34^. In this way, the muscles model is able to take into account their anisotropic behaviour. The scalar Effective Dielectric Permittivity function turns out into a diagonal tensor able to describe the muscle behaviour when excited with an external electric field along any direction. This procedure aims at identifying all parameters in Cole–Cole equation (1) of the muscle excited along the fibers direction to let real and imaginary parts of its Effective Dielectric Permittivity frequency spectra fit the experimental data available in^34^. This plays as a constraint of the following identification problem, stated so that the abovementioned unknown parameters are not “too far” from those of the muscle excited perpendicularly to the fibers. Therefore, the identification problem is stated as:

2 $$\begin{aligned} \begin{array}{l} \min \Vert {\underline{x}}_{L}-{\underline{x}}_{P} \Vert \\ { subject\;to} : {\dot{\varepsilon }}_{eff} (\omega _k,{\underline{x}}_{LD}) - {\dot{\varepsilon }}_{eff_{exp}}(\omega _k)=0 \end{array} \end{aligned}$$

where:

- ${\underline{x}}_{L}$ is the vector of the Cole–Cole parameters of the Effective Dielectric Permittivity when the tissue is excited with an electric field parallel to the fibers,
- ${\underline{x}}_{P}$ is the vector of the Cole–Cole parameters of the Effective Dielectric Permittivity when the tissue is excited with an electric field perpendicular to the fibers,
- $\omega _k$ is the sampling frequency of the experimental data.

**Figure 3:**
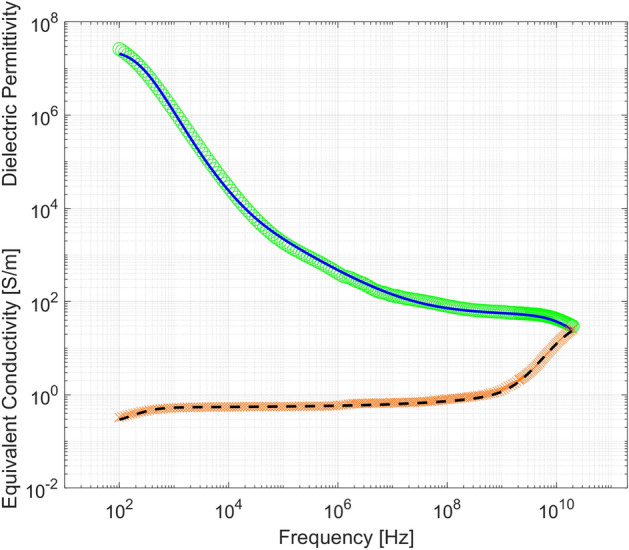
Experimental (circles) vs fitted (solid line) dielectric permittivity and experimental (crosses) vs fitted (dashed line) equivalent conductivity of muscles (excitation parallel to fibers).

The complex equality constraint is a non linear function of the degrees of freedom and the problem is stated as a convex optimization problem whose solution is afforded via the Interior-Point optimization method. Figure 3 shows the results obtained in terms of fitting of the frequency spectra of the dielectric permittivity and equivalent conductivity.

The electrical parameters listed in Table 2 are considered as inputs of the knee model before its optimization and customization. The identified Cole–Cole parameters for the Muscle (Parallel) are reported in bold.

**Table 2:** Parameters of Cole–Cole equation according to equation (2) for Muscles (Parallel) and according to^25^ for other biological tissues.

	$\varepsilon _{\infty }$	$\Delta \varepsilon _{1}$	$\tau _{1}$ [ps]	$\alpha _{1}$	$\Delta \varepsilon _{2}$	$\tau _{2}$ [ns]	$\alpha _{2}$	$\Delta \varepsilon _{3}$	$\tau _{3}$ [$\mu$s]	$\alpha _{3}$	$\Delta \varepsilon _{4}$	$\tau _{4}$ [ms]	$\alpha _{4}$	$\sigma _{dc}$ [S/m]
Dry Skin	4.0	32.0	7.23	0.00	1100	32.48	0.00							0.0002
Wet Skin	4.0	39.0	7.96	0.10	280	79.58	0.00	3.0 × 10^4^	1.59	0.16	3.0 × 10^4^	1.592	0.20	0.0004
Fat	2.5	9.0	7.96	0.20	35	15.92	0.10	3.3 × 10^4^	159.15	0.05	1.0 × 10^7^	15.915	0.01	0.0350
Muscle (Transv.)	4.0	50.0	7.23	0.10	7000	353.68	0.10	1.2 × 10^6^	318.31	0.10	2.5 × 10^7^	2.274	0.00	0.2000
**Muscle (Paral.)**	**4.0**	**50.0**	**10.57**	**0.11**	**7000**	**19.10**	**0.01**	**1.2 × 10**^6^	**725.47**	**0.32**	**2.5 × 10**^7^	**0.737**	**0.00**	**0.2397**
Bone	2.5	18.0	13.26	0.22	300	79.58	0.25	2 × 10^4^	159.15	0.20	2.0 × 10^7^	15.915	0.00	0.0700

### Assumptions

#### Geometry

**Figure 4:**
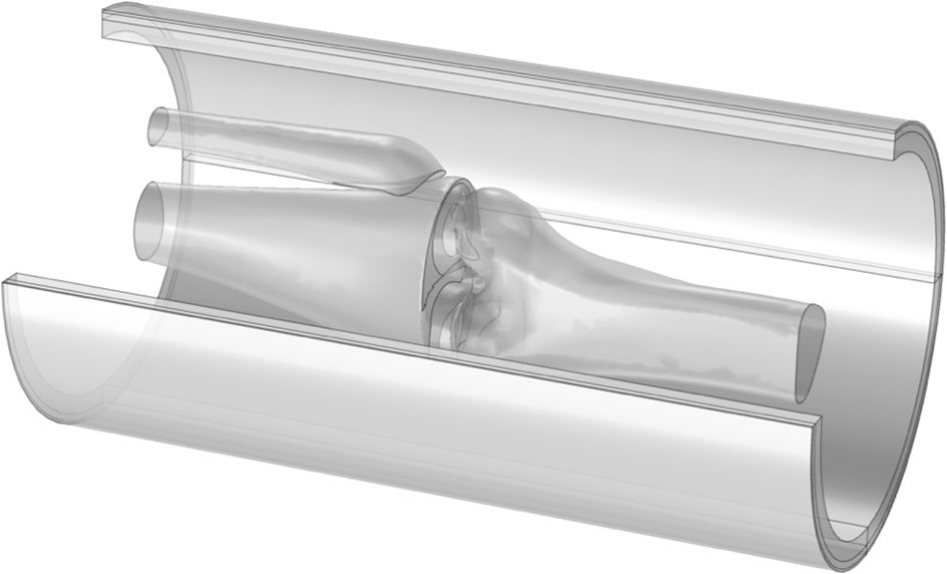
3D structure of human knee.

Three macroscopic knee tissues were considered in the model: skin, muscle, and bone. The bones geometry faithfully reproduced the actual human shape and was inserted inside a cylindiric muscle layer, being the innermost part of a layered structure. The three skin layers, epidermis, dermis, and hypodermis, were partially rearranged. Part of the epidermis was associated with the dermis according to the percentage water content. In fact, epidermis water content ranges between [20–70]% from the stratum corneum to basale^35–37^. Therefore, a structure (Fig. 4) with five layers was realized:

- *Layer 1*: (outermost skin zone) stratum corneum and lucidum, namely *Dry Skin*;
- *Layer 2*: straum granulosum, spinosum, basale, and dermis, namely *Wet Skin*;
- *Layer 3*: hypoderm, subcutaneous fat tissue, namely *Subcutaneous Fat*;
- *Layer 4*: muscle;
- *Layer 5*: bone.

#### Physics

The proposed model of the human knee is based on the numerical solution of the Direct Current formulation of Maxwell equations in the frequency domain and in the presence of lossy dielectric materials (i.e., tissues listed in “Background” section) characterized by relaxation phenomena.

Moreover, the time derivative of the flux density field can be neglected, therefore, Maxwell equations can be simplified into the electric quasi-stationary limit, taking into account simultaneously both a curl-free electric field and the current density field in the tissues.

The current density consists of three contributions: the conduction current generated by ohmic conduction, the Maxwell displacement current equal to the rate of change of the electric displacement field, and the possible external current, not considered here owing to the voltage-driven excitation. Taking into account the presence of dielectric materials by means of their effective dielectric permittivity, the following general formulation holds in terms of scalar electric potential, which turns to be the unknown scalar field of the differential problem:

3 $$\begin{aligned} {\left\{ \begin{array}{ll} -\varepsilon _0{\dot{\varepsilon }}\nabla ^2\overline{\varphi } +\nabla \cdot {\overline{J}}_{ext} = 0 \\ {\overline{\varphi }}|_{\partial \Omega _T}={\overline{\varphi }}_0(\mathbf{r }) \\ -\varepsilon _0{\dot{\varepsilon }}\nabla {\overline{\varphi }}\cdot {\hat{n}}|_{\partial \Omega _S} = J_{n}(\mathbf{r }) \end{array}\right. } \end{aligned}$$

where:

- ${\overline{\varphi }}$ is the phasor of the electric scalar potential;
- ${\overline{\varphi }}_0(\mathbf{r })$ is the value of the Dirichlet boundary condition set on the boundary $\partial \Omega _T$;
- and ${\overline{J}}_{n}(\mathbf{r })$ is the value of the Neumann boundary condition set on the boundary $\partial \Omega _S$.

The Dirichlet boundary conditions set the value of the electric scalar potential across voltage source terminals (labelled as $\Omega _T$). The Neumann boundary conditions set the value of the normal component of the total current across some surfaces. In particular, it imposes natural boundary condition (i.e. electric insulation) overall the skin surface $\Omega _S$.

At last, equations in (3) need to be coupled with additional conditions at interface between two different tissues, stating that the normal component of the current density field is continuous there.

### Personalization

#### Layer thickness customization

An algorithm was conceived to calculate personalized thicknesses of the five knee layers. The input parameters were: sex, Body Mass Index (BMI) and the radius of the mean circumference of knee (*mean-radius*). The radius was obtained as the mean value of three different measurements: at the center of patella, 5 cm above, and 5 cm below. The algorithm consists of the following steps:

- sex-based definition of the thicknesses of *Layer 5* and *Layer 2* according to Table 1 and 3, respectively;
- calculation of the thickness of *Layer 3* (SFT) via linear relation^38^:
  4 $$\begin{aligned} BMI = 0,385\cdot SFT + 16,991; \end{aligned}$$
- computation of the thickness of *Layer 4* as the difference between the *mean-radius* and the thicknesses of other layers, having constrained *Layer 1* equal to 60 $\mu$m both for males and females^20,39,40^.

An example of thicknesses [mm] of model layers for males and females, assuming Body Mass Index (BMI) equal to 23 kg$\setminus$m^2^ and radius of mean circumference equal to 6.5 cm, is reported in Table 3.

**Table 3:** Thicknesses [mm] of model layers for males and females (BMI = 23 kg/m^2^, and radius of mean circumference = 6.5 cm).

	Layer 1	Layer 2	Layer 3	Layer 4	Layer 5
	Dry Skin	Wet Skin	Subcutaneous Fat	Muscle	Bone
Male	0.060	4.501	15.192	15.621	25.640
Female	0.060	3.666	16.971	17.552	22.760

#### Dielectric parameters customization

The customization of the knee-model electrical behavior was realized by adapting reference values in Table 2 for fitting individual experimental data. To this aim, the parameters most affecting the bioimpedance magnitude spectrum were determined by a sensitivity analysis. However, considering their large number (almost eighty), a reduction of the parameters is needed to carry out the customization process at an affordable computational burden.

First, the number of the parameters in Eq. (1) to be optimized for each tissue was reduced by assessing the frequency range and the effects of each relaxation on the spectrum of the actual dielectric permittivity of each tissue. Then, based on data collected in a preliminary experimental campaign, a one-at-a-time-parameter sensitivity analysis was carried out in order to collect the parameters most affecting the bioimpedance magnitude spectrum. The resulting seven Cole–Cole parameters most impacting on the simulated bioimpedance magnitude are reported in Table 4.

**Table 4:**
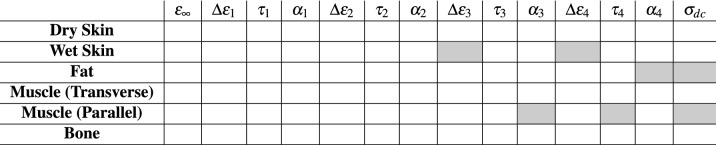
The seven parameters used for personal model customization.

At last, a Nelder-Mead simplex optimization procedure is used for identifying the actual value of the extracted seven parameters in order to fit the individual bioimpedance magnitude spectrum within a given tolerance. The prediction error is assessed in terms of relative difference between reconstructed (*Z*_*sim*_)and experimental (*Z*_*exp*_) bioimpedance magnitude:

5 $$\begin{aligned} \begin{aligned} \varepsilon _{fit} = \frac{\Vert Z_{exp}(\omega _k)-Z_{sim}(\omega _k) \Vert }{\Vert Z_{exp}(\omega _k)\Vert } \end{aligned} \end{aligned}$$

where $\omega _k$ is the experimental angular frequency.

## Experimental validation

An experimental campaign was carried out by in-vivo impedance spectroscopy measurements for validating the knee model customization procedure. Thirty volunteers (27% male and 73% female, average age 36±15 years, mean knee circumference 39 cm ± 4 cm, and BMI 25±4 kg/m^2^) were included in this study. Ten volunteers were affected by osteoarthritis with Kellgrenn–Lawrence^41^ grade spanning over the range [1,4]. The remaining twenty volunteers exhibited Kellgrenn–Lawrence grade equal to zero. All the participants did not present swelling of the knee, were not affected by skin diseases, and did not report previous joint surgeries, nor other kind of pathology. Measurements were performed in accordance with the relevant guidelines and regulations. All the experimental protocols were approved by the ethics committee of the “Giovanni Paolo II” scientific research, hospitalization and healthcare institute (IRCSS) of Bari (Italy). “Giovanni Paolo II” ethics committee was territorial competent for IRCSS Maugeri—Cassano delle Murge: the partner of University Federico II for this research. Informed consent containing all the information on the experiment was provided and signed by all the participants.

### Experimental setup

**Figure 5:**
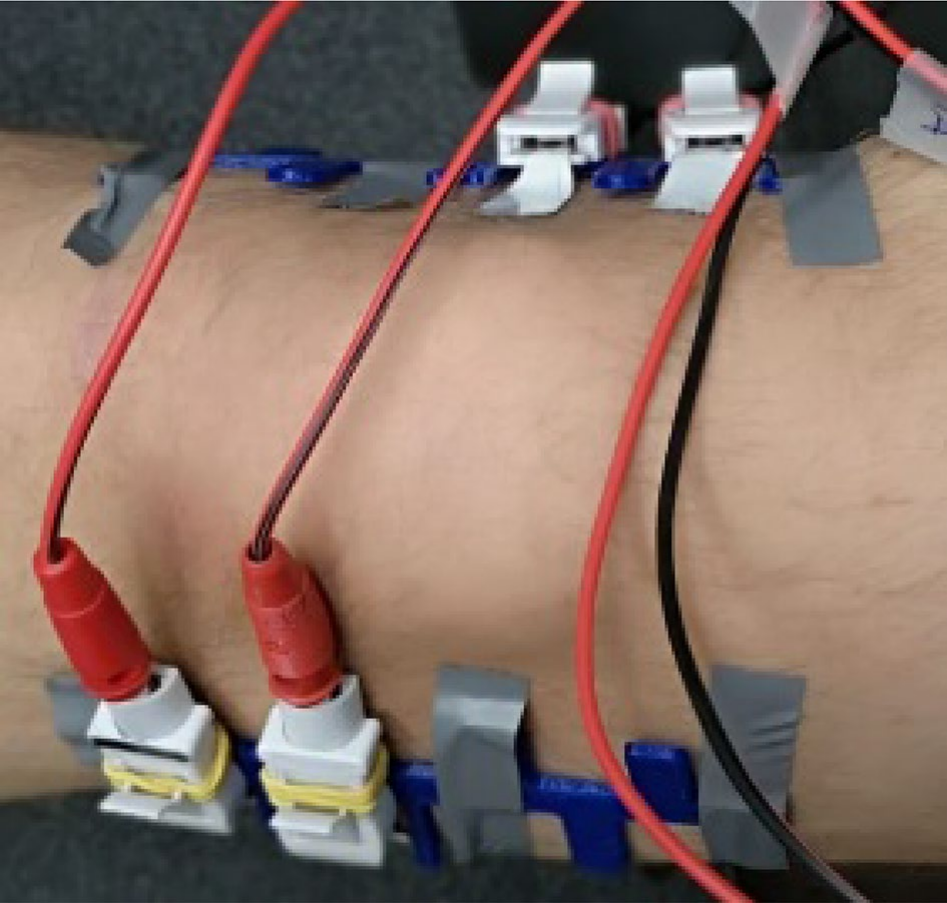
Four-wire electrodes applied to the knee opposite sides.

Measurements were carried out by means of the Drug Under Skin Meter^10^ for exploring thirteen frequency values in the range [1.00–49.00] kHz in compliance with safety regulations (IEC-60601 standard). Volunteers were completely insulated from the ground by means of a rubber mat. A battery-powered laptop gathers the data collected by the instrument. Effects of parasitic capacitances are minimized thanks to the equipotential connection between the patient and the instrument chassis realized by using an Electrocardiography clamp electrode, positioned on the right ankle. DUSM was powered by a USB cable from the laptop-PC battery. Therefore, the device was never directly connected to the main supply. Measurements were carried out by pre-gelled cutaneous electrodes (Fiab P500) using a four-wire configuration. The laboratory temperature and humidity were registered. Preliminarily, the patient parameters such as sex, age, measure of knee circumferences and body mass index (BMI) were recorded. The circumference of knee were measured for the correct electrodes positioning as shown in Fig. 5.

### Impedance spectroscopy measurements and optimization results

**Figure 6:**
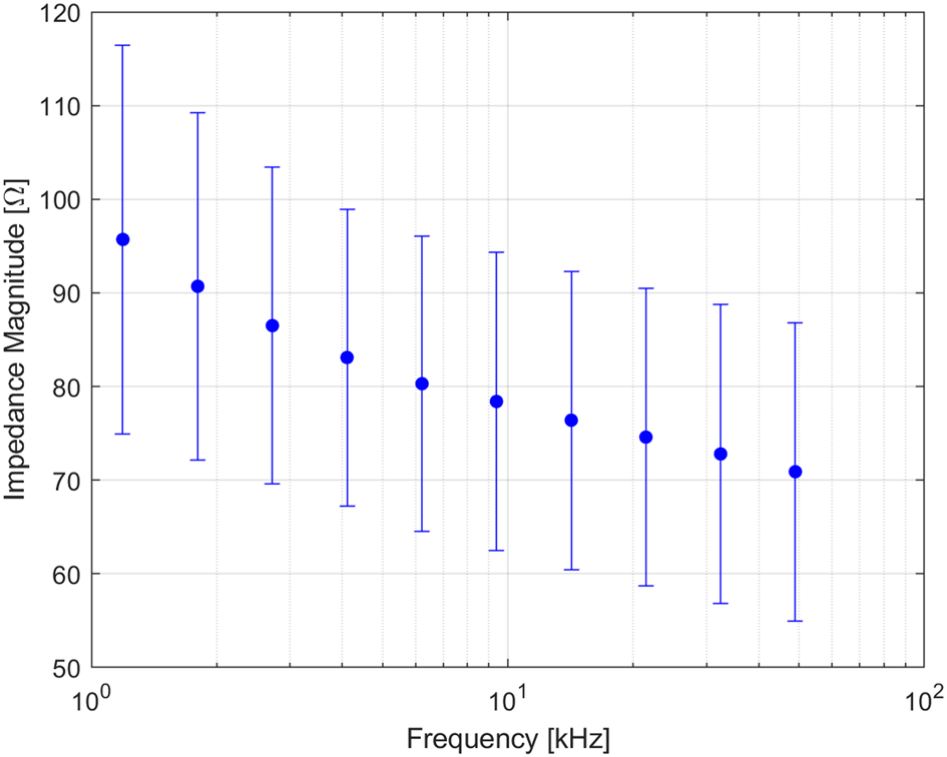
Impedance magnitude spectroscopy: mean (dots) and 1-sigma-reproducibility (errorbars) on all the 30 subjects.

DUSM impedance spectroscopy results are reported in Fig. 6 in terms of mean magnitude value at each frequency and errorbars of 1-sigma-reproducibility on all the subjects. High percentage values of standard deviation with respect to the mean values (22% at low frequencies and 16% at high frequencies) revealed a significant inter-individual variability. In particular, ANOVA revealed a pathology condition not impacting on impedance trend (p-value < 0.002), by confirming the literature results.

**Figure 7:**
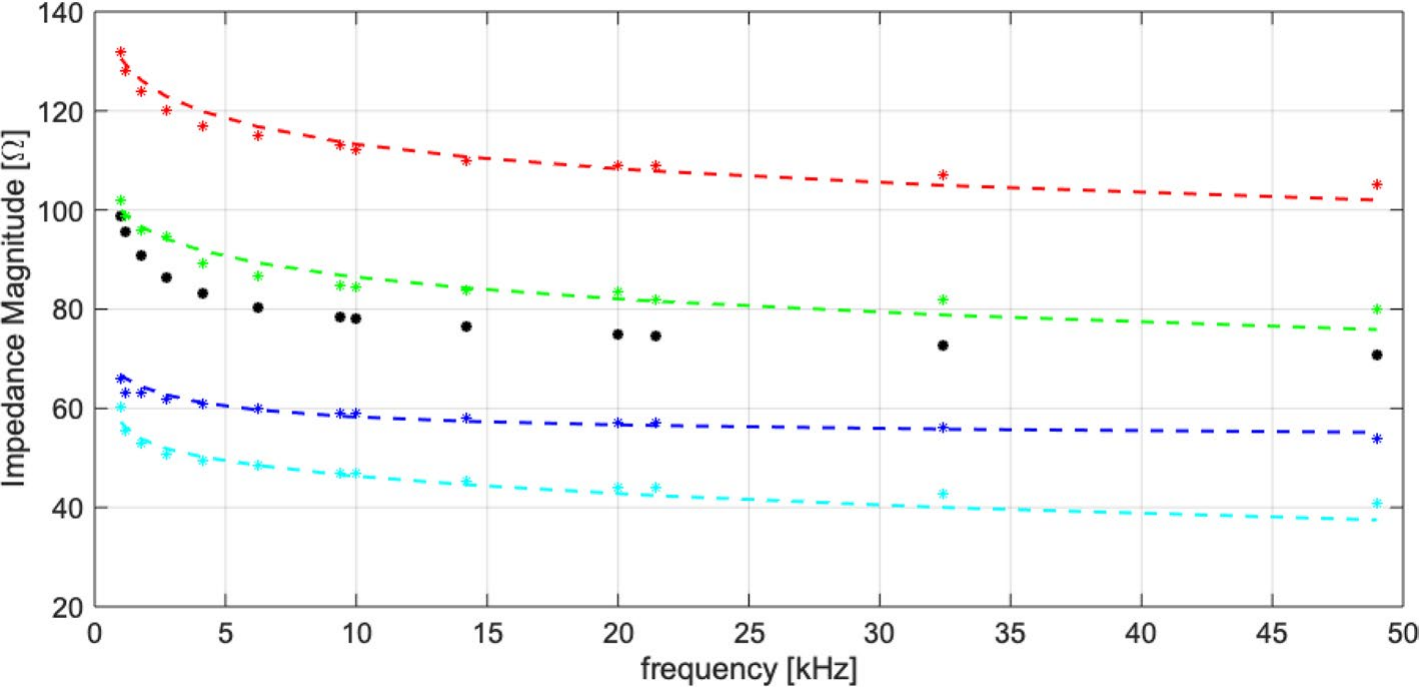
Impedance magnitude identification on arthrosic and healthy significant four subjects: average impedance on thirty subjects (black dotted line). Stars: experimental data; dashed line: fitting models. Healthy male subject (M$_H$): blue. Healthy female subject (F$_H$): red. Arthrosic male subject (M$_A$): cyan. Arthrosic female subject (F$_A$): green.

Among the thirty subjects, the optimization procedure achieved a worst-case reconstruction error lower than 5% and an average error of 3%. In Fig. 7, results about four out of the thirty volunteers are shown. Two osteoarthritic volunteers were chosen at the extremes of Kellgren–Lawrence scale, namely of grades 1 and 4. The others were chosen with the highest and the lowest average bioimpedance magnitude among healthy people. The optimization returns the individual impedance spectroscopy curve fitting within a reconstruction error in 1–4%. For each individual, the parameters of Table 4 were optimized, as reported in Table 5.

**Table 5:** Optimized value of Cole–Cole parameters for Arthrosic and Healthy individuals.

		Healthy		Arthrosic	
		M$_H$	F$_H$	M$_A$	F$_A$
Wet Skin	$\Delta \varepsilon _{3}$	5000	5085	15,000	30,000
	$\Delta \varepsilon _{4}$	75,000	5000	15,000	30,000
Fat	$\alpha _{4}$	0.200	0.240	0.436	0.250
	$\sigma _{dc}$ [S/m]	0.052	0.032	0.167	0.079
Muscle (Parallel)	$\alpha _{3}$	0.320	0.313	0.100	0.325
	$\tau _{4}$ [ms]	5.896	5.421	0.002	0.737
	$\sigma _{dc}$ [S/m]	0.224	0.015	0.200	0.240

The results of the optimization proved the feasibility of personalizing the knee model to the specific characteristics of individuals. Seven parameters were good enough to keep the maximum error below 5%. Three parameters belonged to the parallel component of the muscle layer identified through this study.

## Conclusion

A personalizable FEM model of knee for improving reproducibility in NSAIDs transdermal delivery measurement was presented. The model geometry consists of five layers: bone, muscle, subcutaneous fat, wet skin and dry skin. Thicknesses of layers were personalized according to sex, BMI index, and mean circumference of knee. Electrical characterization of the tissues took into account their non ideal lossy and dispersive behavior by means of Cole–Cole model. Muscle tissue was characterized anisotropically: the parameters of the Cole–Cole equation of the parallel component were calculated from experimental data available in the literature. Personalized models were identified by tuning seven electrical parameters: less than 10% of the initial amount. Models were validated on thirty volunteers, twenty healthy and ten affected by knee osteoarthritis (Kellgren–Lawrence grade [1,4]). The average reconstruction error was 3% and lower than 5% in the worst case. The customization of a simplified knee FEM model with a low computational burden was proven to be feasible both for healthy and osteoarthritic knees. A forthcoming modeling of NSAIDs electrical behaviour in human tissues (*Drug-in-Knee Model*) combined with the personalized models will allow high reproducible assessment of drug amount transdermally delivered.
